# Semen and serum platinum levels in cisplatin‐treated survivors of germ cell cancer

**DOI:** 10.1002/cam4.4480

**Published:** 2021-12-17

**Authors:** Eoghan R. Malone, Jeremy Lewin, Xuan Li, Wen‐Jiang Zhang, Susan Lau, Keith Jarvi, Robert J. Hamilton, Aaron R. Hansen, Eric X. Chen, Philippe L. Bedard

**Affiliations:** ^1^ Division of Medical Oncology and Hematology Princess Margaret Cancer Centre University Health Network Department of Medicine University of Toronto Toronto Ontario Canada; ^2^ Division of Urology University of Toronto Toronto Ontario Canada; ^3^ Murray Koffler Urologic Wellness Centre Mount Sinai Hospital Joseph and Wolff Lebovic Health Complex Toronto Ontario Canada

**Keywords:** chemotherapy toxicity, cisplatin, fertility, survivorship, testicular cancer

## Abstract

**Background:**

Testicular cancer survivors often have impaired gonadal function possibly related to chemotherapy. Platinum is a heavy metal that can be detected at low levels in serum many years after treatment, it is not known whether platinum also persists in semen and if platinum persistence in semen is associated with impaired fertility.

**Methods:**

Adult cisplatin‐treated testicular cancer survivors were enrolled. High‐Performance Liquid Chromatography‐tandem mass spectrometry was used to measure semen and serum platinum levels. Semen quality and DNA Fragmentation Index (DFI) were assessed.

**Results:**

From 11/2017 to 12/2019, 38 patients (median age 32 years; range: 19–52) were enrolled. Median cumulative cisplatin dose was 301 mg/m^2^ (range: 274–404). Platinum levels were higher in semen than in blood (*p* = 0.03). Semen platinum levels were not significantly associated with time from last cisplatin dosing (*r* = −0.34; *p* = 0.09) nor cumulative dose (*r* = −0.10, *p* = 0.63). Sperm concentration was correlated with time from last cisplatin dosing (*r* = 0.58, *p* < 0.001) but not with semen platinum level (*r* = −0.15, *p* = 0.46). DFI was not significantly associated with time from last cisplatin dosing (*r* = 0.55, *p* = 0.08) or semen platinum level (*r* = −0.32, *p* = 0.33). In four patients with serial semen samples, platinum level decreased and sperm concentration and motility increased over time.

**Conclusions:**

Platinum is detected in semen of testicular cancer survivors at higher levels than matched blood samples. These preliminary findings may have important implications for the reproductive health of survivors of advanced testicular cancer, further study is needed to assess the relationship between platinum persistence in semen and recovery of fertility postchemotherapy.

## Graphical abstract

Platinum is a heavy metal that can be detected at low levels in serum many years after treatment, it is not known that whether platinum also persists in semen and if platinum persistence in semen is associated with impaired fertility. Platinum is detected in semen of testicular cancer survivors at higher levels than matched blood samples. These preliminary findings may have important implications for the reproductive health of survivors of advanced testicular cancer, further study is needed to assess the relationship between platinum persistence in semen and recovery of fertility postchemotherapy.

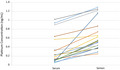

## 1 BACKGROUND

Cisplatin is widely used in the treatment of various malignancies such as advanced testicular cancer, gastroesophageal cancer, head and neck cancer, lung cancer, and osteosarcoma. Germ cell tumors (GCTs) are the most common solid malignancies diagnosed in Adolescent and Young Adult (AYA) males.^1^ Even for patients with metastatic disease, cure rates remain high with modern multimodality cisplatin‐based chemotherapy and surgical resection for residual masses for patients with nonseminoma histology. An important survivorship imperative is to minimize side effects, particularly those long‐term effects of cisplatin without compromising efficacy. Many studies have shown that GCT survivors have increased incidences of late effects such as ototoxicity, neuropathy, nephropathy, cardiovascular disease, and metabolic dysregulation postcisplatin‐based chemotherapy.^2^ The underlying mechanisms for these late effects remain obscure but may be related to chronic tissue damage induced by circulating platinum.^3^, ^4^, ^5^

Platinum is a heavy metal that can be detected in a variety of organs such as serum, fat tissue, bone, liver, kidney, and lungs after cisplatin administration.^6^, ^7^, ^8^, ^9^ Low levels of circulating platinum can be detected up to 20 years after cisplatin chemotherapy, likely due to release from regenerating tissues, where platinum is stored.^10^ More recently, long‐term circulating platinum levels have been correlated with the development and severity of ototoxicity and neurotoxicity.^3^, ^4^ Limited studies have focused on the relationship between circulating platinum levels and peripheral neuropathy in GCT patients.^4^, ^11^ Higher serum platinum levels are related to more severe long‐term neuropathy in these patients,^4^ which is consistent with high concentrations of platinum identified in the dorsal root ganglia in postmortem studies.^12^

It is well known that testicular cancer survivors may experience impaired gonadal function secondary to treatment or as a predisposing biologic factor related to the development of testicular cancer. Impairment of Leydig cell function is seen prior to orchidectomy^13^ with a high prevalence of abnormal spermatogenesis identified in the contralateral testicle for patients with unilateral GCT. Chemotherapy can induce dose‐dependent spermatogenic impairment and Leydig cell dysfunction.^14^ In one study of 170 patients with GCT who received platinum‐based therapy, only 64% were normospermic at 30 months posttreatment.^15^ In addition, several other groups have identified testicular dysfunction in patients treated with cytotoxic chemotherapy as documented by sperm count reduction or raised Follicle Stimulating Hormone (FSH).^15^, ^16^ Although Leydig cells are relatively resistant to cytotoxic chemotherapy, persistent LH elevation and testosterone reduction have been documented in testicular cancer patients for reasons that are largely unknown.^17^, ^18^, ^19^

We hypothesized that platinum would be detectable in semen samples from testicular cancer survivors and levels of semen cisplatin would be associated with impaired spermatogenesis. Using a High‐Performance Liquid Chromatography‐Mass Spectrometry (HPLC/MS‐MS) method developed to quantitate platinum in both serum and semen, we evaluated circulating and semen platinum levels in testicular cancer survivors along with markers of gonadal function and semen quality.

## 2 METHODS

### 2.1 Patient selection and blood sample collection

Male GCT patients aged 18 years and older who were previously treated with three to four cycles of cisplatin‐based chemotherapy as the initial treatment of metastatic GCTs were prospectively identified and enrolled. Venous blood samples were obtained and platinum levels were determined as were serum tumor markers (Alpha Fetoprotein, beta Human Chorionic Gonadotropin, Lactate Dehydrogenase), FSH, Leteinizing Hormone (LH), testosterone, estradiol, and creatinine. Information about primary tumor site, histology, International Germ Cell Cancer Collaborative Group (IGCCCG) risk classification, and timing of prior chemotherapy were collected through chart review using standardized case report forms. This study was approved by the institutional research ethics board and conducted according to the Declaration of Helsinki. All patients provided written consent prior to participating in the study.

### 2.2 Semen analysis

Semen samples were collected according to standard clinical protocols.^20^ Each semen sample was examined by a light microscope according to World Health Organization criteria (2010) for total sperm count, total motility (%), and morphology (% abnormal forms). In addition, sperm DNA damage was assessed by DNA fragmentation (DNA Fragmentation Index, DFI). Patients were contacted by a specialized nurse to review sperm testing results (as per standard of care) but were not informed of platinum levels in serum or semen. Depending on results follow‐up visits and further semen sampling were organized per standard of care. Follow‐up semen samples were also analyzed for platinum levels if there was a sufficient sample available.

### 2.3 Serum and semen platinum analysis

Venous blood samples (5 ml) were taken from the antecubital fossa via venipuncture and immediately centrifuged at 1300 × g for 10 min at 4°C. Serum was separated and transferred to cryogenic vials. These cryogenic vials were labeled with anonymized patient identifiers and times of blood draw. The laboratory personnel was blinded to patient characteristics when performing analysis of platinum levels in serum and semen samples. Cryogenic vials were stored in a −80°C freezer within 30 min of collection until analysis. Platinum levels in serum and semen were quantified with an and blood work results HPLC‐MS/MS method as described previously.^21^

### 2.4 Statistical analysis

Summary statistics such as means, medians, standard deviations, ranges, frequencies, and proportions were reported to describe patient characteristics, semen, and serum platinum levels, and blood work results. Pearson correlations and corresponding *p* values were calculated to evaluate the pairwise correlations among relevant covariates, platinum levels, and sperm quality. Linear regression models were used to evaluate the association between time from the last cisplatin‐based chemotherapy, and blood work results with platinum levels. All statistical analyses were conducted using R version 3.6.1 and Microsoft Excel.

## 3 RESULTS

### 3.1 Clinical characteristics

**FIGURE 1:**
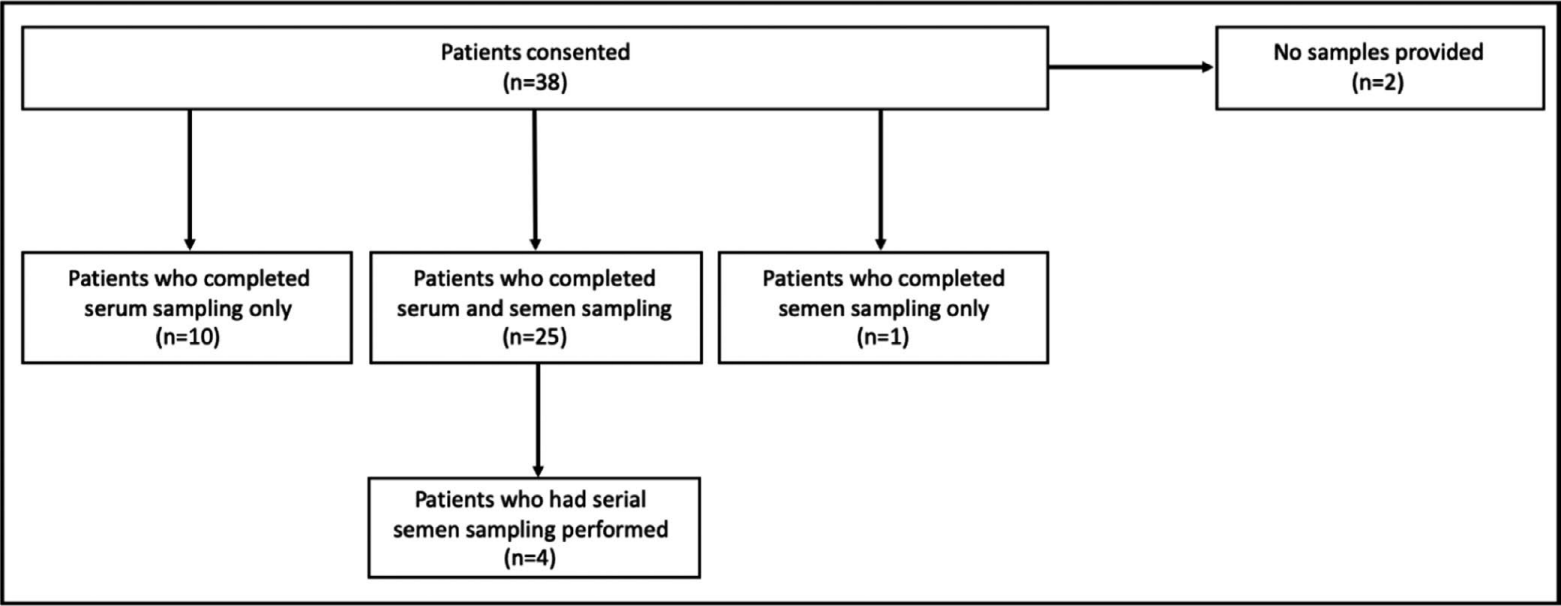
Consort diagram

Between November 2017 and December 2019, 38 patients were prospectively enrolled (Figure 1). Baseline characteristics of the patients are summarized in Table 1. Patients had a median age of 32 years (range: 19–52 years), 95% of patients had a testicular primary tumor with 5% mediastinal, 68% were nonseminoma, and 32% were seminoma. Thirty‐one patients (82%) were treated with three cycles of bleomycin, etoposide, and cisplatin. The median body surface area was 2.03 m^2^ (range: 1.81–2.61) and the median cumulative cisplatin dose was 301 mg/m^2^ (range: 274–404) (Table 1).

**TABLE 1:** Baseline demographics and clinical characteristics (*N* = 38)

Parameter	Number (%)
Age	
Median (range)	32 (19, 52)
Histology	
Seminoma	12 (32)
Nonseminoma	26 (68)
Primary Tumor Site	
Testicle	36 (95)
Mediastinum	2 (5)
IGCCCG Risk Category	
Good	31 (82)
Intermediate	5 (13)
Poor	2 (5)
Chemotherapy	
BEPx3	31 (82)
BEPx3 + EPx1	3 (8)
VIPx4	2 (5)
BEPx4	1 (2.5)
EPx4	1 (2.5)
Total cisplatin dose/m^2^	
Median (range)	301 (274, 404)
Body surface area	
Median (range)	2.03 (1.81, 2.61)
Time from completion of chemotherapy to blood collection (months)	
Median (range)	10.8 (0.5, 36.4)
Time from completion of chemotherapy to semen collection (months)	
Median (range)	14.1 (1.4, 39.8)

Abbreviations: BEP, bleomycin, etoposide, cisplatin; EP, etoposide, cisplatin; VIP, etoposide, ifosfamide, cisplatin.

### 3.2 Platinum levels

**FIGURE 2:**
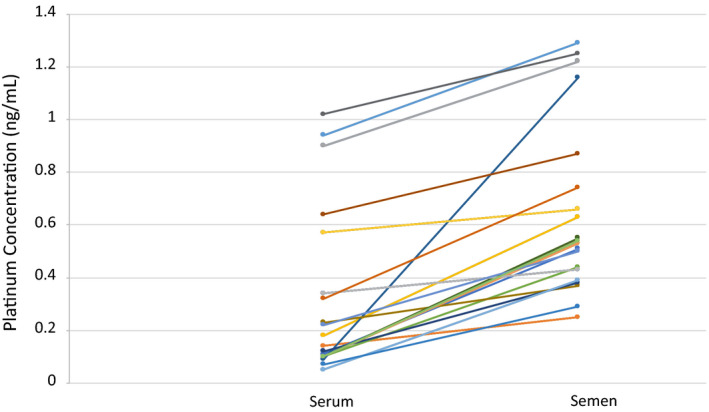
Comparison of paired serum and semen platinum levels

**FIGURE 3:**
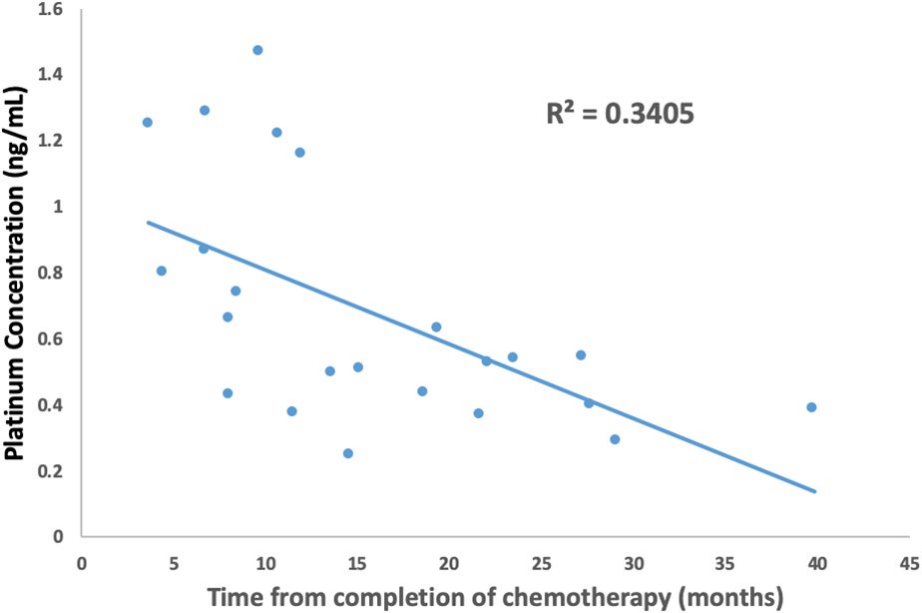
Correlation between time from completion of chemotherapy and semen platinum levels

Platinum was detectable in 86% of serum samples. The median serum platinum concentration was 0.1 ng/ml (range: 0–22.6 ng/ml) at a median of 11 months (range 0.5–36 months) postcompletion of treatment. Platinum was detectable in 100% of semen samples. The median semen platinum concentration was 0.5 ng/ml (range: 0.2–28.7 ng/ml) at a median of 14 months (range: 1.3–40 months) posttreatment completion (Table 2). There were five serum samples in which platinum was undetectable, three of these had paired semen samples and in every case, platinum was detectable in the semen sample. Platinum levels were found to be higher in semen than in paired serum samples in all cases, even though semen samples were usually collected several months after blood collection (*p* = 0.03) (Figure 2). The median ratio of semen to serum platinum was 2.31 (range: 1.16–12.89). There was a trend toward higher semen platinum levels with a shorter time from the last cisplatin treatment (*r* = −0.34, *p* = 0.09) (Figure 3), but there was no association between semen platinum level and cumulative cisplatin dose (*r* = −0.10, *p* = 0.63). Sperm concentration was correlated with the time interval from last cisplatin dosing (*r* = 0.58, *p* < 0.001) but not with semen platinum level (*r* = −0.15, *p* = 0.46) (Figures S2 and S3). For four patients with serial semen samples, the semen platinum level decreased with time while sperm concentration and motility increased in each patient. The median time between the two serial samples was 6.5 months (range: 5.9–9.2 months) (Figure S1). Regression analysis of these four paired samples showed that on average, the semen platinum level decreased by 0.003 ng/ml/day (95% CI −0.01 to 0.005, *p* = 0.52).

**TABLE 2:** Comparison of semen and serum platinum concentrations and time of sampling

	Median time of sampling in months (range)	Median platinum concentration in ng/ml (range)	Standard deviation	Samples with detectable platinum (%)
Serum (*n* = 35)	10.8 (0.5–36.4)	0.1 (0–22.6)	3.9	86
Semen (*n* = 26)	14.1 (1.4–39.8)	0.5 (0.2–28.7)	5.5	100

### 3.3 Baseline demographics and laboratory investigations

Covariate analysis of age, creatinine, FSH, LH, testosterone, and estradiol with serum and semen platinum levels are displayed in Table 3. Only the testosterone level was significantly associated with both the serum and semen platinum levels indicating that patients with higher testosterone levels also had higher serum/semen platinum levels.

**TABLE 3:** Univariate regression analyses between covariates and serum and semen platinum levels

Covariate	Serum		Semen	
	Estimate (95% CI)	*p* value	Estimate (95% CI)	*p* value
Age	0.03 (−0.14, 0.19)	0.77	0.04 (−0.25, 0.32)	0.8
Creatinine	−0.05 (−0.14, 0.04)	0.27	−0.06 (−0.21, 0.09)	0.43
FSH	0.05 (−0.13, 0.23)	0.59	0.0087 (−0.27, 0.29)	0.95
LH	0.02 (−0.51, 0.56)	0.93	−0.05 (−0.89, 0.8)	0.92
Testosterone	0.22 (0.01, 0.43)	0.04	0.44 (0.09, 0.79)	0.015
Estradiol	0.006 (−0.04, 0.05)	0.8	0.0072 (−0.08, 0.01)	0.88

### 3.4 DNA fragmentation

A DFI of <20% is considered to have a good chance of achieving natural pregnancy, whereas a DFI of >20% is associated with a reduced likelihood of natural pregnancy even with other sperm parameters (sperm count, motility) being normal. The median DFI was 4.75% (range: 0.85%–17.7%) and improved with time from completion of chemotherapy. The DFI was not associated with time from last cisplatin dosing (*r* = 0.55, *p* = 0.08) or semen platinum level (*r* = −0.32, *p* = 0.33) (Figure S4). DFI was measured in three of the four patients with serial semen samples and the DFI increased over time. In cases where serial samples were collected the DFI was 0.90% (range: unrecordable–1.62%) at time point 1, and 5.5% (range: 2.76–7.23%) at time point 2.

## 4 DISCUSSION

Previous studies have shown that the persistence of platinum in serum is correlated with the severity of long‐term adverse effects in testicular cancer survivors. Since cisplatin is widely used to treat many cancers that affect adolescents and young adults, such as HPV‐associated squamous cell head and neck cancer, osteosarcoma, and ovarian cancer, it is highly likely that other long‐term cancer survivors who were treated with cisplatin will experience similar long‐term side effects due to platinum persistence. Our study is the first study to demonstrate that platinum can be detected in semen from men treated with cisplatin‐based chemotherapy. Platinum was detectable in semen in all patients up to 40 months following the completion of cisplatin‐based chemotherapy. Furthermore, semen platinum levels are on average 2.3‐fold higher than serum platinum levels despite the fact that semen samples were collected several months after blood samples. The finding suggests that the testicular parenchyma is a reservoir for tissue‐bound platinum following chemotherapy treatment. However, semen platinum levels decrease over time, and this decrease in semen platinum levels is associated with improved sperm count and quality. It is not clear whether the decrease in semen platinum directly leads to the improvement in sperm count and quality.

The findings from this study can be used to inform evidence‐based counseling for fertility planning for testicular cancer survivors. There is currently no consensus regarding the optimal timing to father a child for cancer survivors after chemotherapy treatment. The American Society of Clinical Oncology recommends a waiting period of 2–5 years after treatment completion,^22^ whereas the Society for the Study of Male Reproduction recommends waiting 1–2 years.^23^ However, it is commonly assumed that healthy sperm production would start within 2 years of completion of chemotherapy. Other studies have evaluated sperm integrity and spermatogenesis in testicular cancer patients pre‐ and posttreatment with serial semen sampling. Reduced total sperm count (up to 1 year) and motility (up to 9 months) from baseline in the initial period posttreatment before recovery began with a return to near baseline values by 2 years after treatment. DFI analysis in these survivors demonstrated that sperm DNA damage was greatest in the first 6 months after treatment.^24^ Our results are consistent with these findings and demonstrate that the semen platinum levels are highest in the immediate period postchemotherapy and decrease over time. However, it is unknown whether the lack of recovery of sperm production is related to higher levels of platinum in semen.

There are several limitations of our study. Although testicular cancer is the most common cancer diagnosed in the AYA male population, it is a relatively rare cancer in the general population (estimated 1150 men diagnosed in Canada annually)^25^ and we enrolled 38 men over a 2‐year period from a single academic center. Blood samples were collected on the date that patients were seen in the clinic and consented to participate in the study. However, due to logistical issues, semen samples were collected at a different date and required a return appointment at a nearby fertility assessment center. As a result, there was a time lag between serum and semen collections for platinum analysis. Precisplatin chemotherapy semen samples were not available for comparative analysis. It has previously been shown that platinum was detected in serum samples from men not previously treated with cisplatin‐based chemotherapy, this is possibly related to platinum group element bio‐accumulation related to the use of catalysts for motor vehicle exhaust, etc.^26^ It would be of interest to analyze the platinum concentration in semen samples collected prior to the administration of cisplatin or from a cohort of testicular cancer patients who were not treated with chemotherapy. Finally, the follow‐up for this prospective cohort is short, and only four serial semen samples were available for longitudinal analysis and there is no data yet available regarding successful conception following treatment.

It is important to note that patients that develop testicular cancer tend to have lower rates of fertility. In recent times, there has been an increase in the rates of hypospadias, cryptorchidism, impaired spermatogenesis, and testicular germ cell cancer which may collectively signify testicular dysgenesis syndrome.^27^, ^28^ We plan to expand our study to include cisplatin‐treated AYA patients with other types of cancer to shed further light on the impact of cisplatin‐based chemotherapy on fertility without a testicular dysgenesis backdrop.

This study is the first to show that platinum can be detected in semen samples of patients treated with cisplatin‐based chemotherapy, semen platinum can be detected up to 40 months postcompletion of chemotherapy, and levels decrease over time. The effect of platinum persistence in semen on long‐term fertility in testicular cancer survivors is unknown. We plan to continue to enroll patients and to expand to include AYA male patients receiving cisplatin‐based chemotherapy for other types of cancer such as sarcoma and head and neck cancer. We will also examine archival tissue from patients who have had an orchiectomy after cisplatin‐based chemotherapy to identify where platinum is bound within the testicular parenchyma. Where available, stored prechemotherapy will also be reviewed to assess sperm characteristics pre‐ and postchemotherapy. These ongoing studies are needed to better understand the relationship between platinum persistence and recovery of fertility following cisplatin‐containing chemotherapy.

## CONFLICT OF INTEREST

PLB reports grants from AstraZeneca, BMS, GSK, Sanofi, Lilly, Pfizer, Roche/Genentech, Novartis, Merck, SeaGen, Nektar Therapeutics, Servier, SignalChem, PTC Therapeutics, Mersana, and Amgen, outside the submitted work; and uncompensated advisory boards with BMS, Merck, SeaGen, Sanofi, Lilly, Amgen, and Roche/Genentech. ARH reports grants from Genetech/ Roche, grants and personal fees from Merck, GlaxoSmithKline, and grants from Bristol‐Myers Squibb, Novartis, Boehringer‐Ingelheim, Boston Biomedical, AstraZeneca, and MedImmune. The remaining authors have no conflicts of interest to declare.

## AUTHOR CONTRIBUTION

Concept and design: ERM, JL, KJ, RJH, ARH, EXC, PLB; Financial support: N/A; Collection and assembly of data: ERM, JL, WJZ, SL, ARH, EXC, PLB; Data analysis and interpretation: ERM, XL, EXC, PLB; Manuscript writing: All authors; Final approval of manuscript: All authors.

## ETHICS STATEMENT

This study was approved by the institutional research ethics board and conducted according to the Declaration of Helsinki. All patients provided written consent prior to participating in the study.

## Supporting information

Fig S1‐S4

## Data Availability

Data are available on request from the authors.
