# Supplementary material for: Semen and serum platinum levels in cisplatin‐treated survivors of germ cell cancer
Source: Cancer Med. 2021 Dec 17;11(3):728–34. doi: 10.1002/cam4.4480 (PMC8817086; doi:10.1002/cam4.4480)

**Supplementary Figure 1:** Plot of the four patients with serial semen sampling performed showing semen platinum levels decreasing with time

**
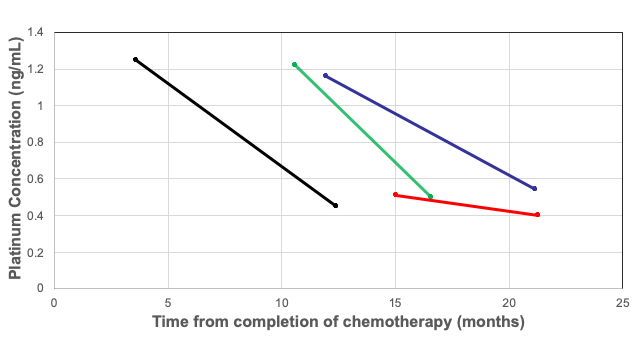
**

**Supplementary Figure 2:** Correlation between semen platinum concentration and sperm concentration


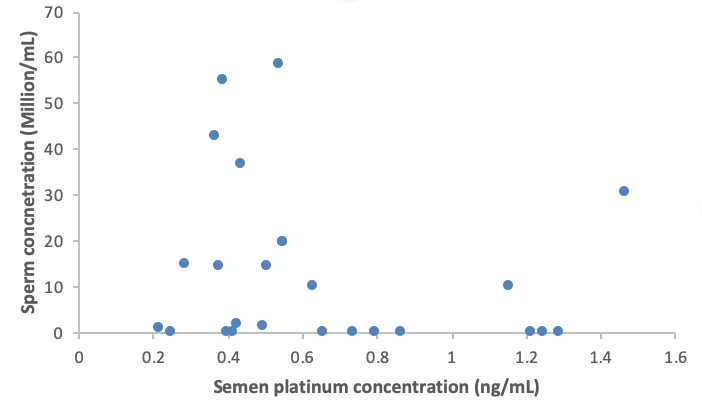


**Supplementary Figure 3:** Correlation between semen platinum concentration and sperm motility


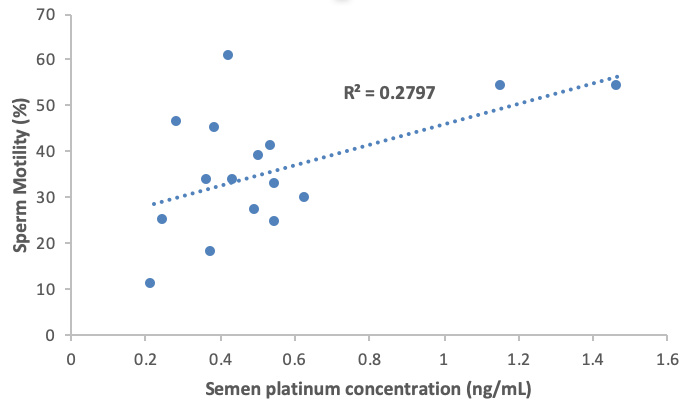


**Supplementary Figure 4:** Correlation between semen platinum concentration and DNA fragmentation index


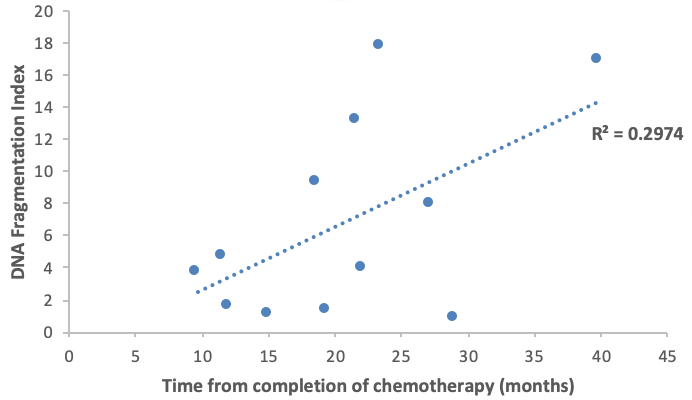

Supplement: Supplementary file 1 — Fig S1‐S4 [file CAM4-11-728-s001.docx]
